# Does the reason for discontinuation of a first TNF inhibitor influence the effectiveness of a second TNF inhibitor in axial spondyloarthritis? Results from the Swiss Clinical Quality Management Cohort

**DOI:** 10.1186/s13075-016-0969-2

**Published:** 2016-03-22

**Authors:** Adrian Ciurea, Pascale Exer, Ulrich Weber, Giorgio Tamborrini, Beate Steininger, Rudolf O. Kissling, Jürg Bernhard, Almut Scherer, on behalf of the Rheumatologists of Swiss Clinical Quality Management Program for Axial Spondyloarthritis

**Affiliations:** Department of Rheumatology, University Hospital Zurich, Gloriastrasse 25, CH-8091 Zurich, Switzerland; Private Rheumatology Practice, Basel, Switzerland; King Christian 10th Hospital for Rheumatic Diseases, Gråsten, Denmark; South Jutland Hospital, Denmark Institute of Regional Health Research, University of Southern Denmark, Odense, Denmark; Department of Rheumatology, Bethesda Hospital, Basel, Switzerland; Department of Rheumatology, Balgrist University Hospital, Zurich, Switzerland; Department of Rheumatology and Rehabilitation, Bürgerspital, Solothurn, Switzerland; Swiss Clinical Quality Management Foundation, Zurich, Switzerland

**Keywords:** Axial spondyloarthritis, Tumor necrosis factor inhibitors, Switching, Drug retention

## Abstract

**Background:**

With regard to switching tumor necrosis factor inhibitors (TNFi) in axial spondyloarthritis (axSpA), conflicting results have been reported as to whether the effectiveness of a second TNFi depends on the reason for discontinuation of the first TNFi.

**Methods:**

Patients with a clinical diagnosis of axSpA starting a second TNFi in the Swiss Clinical Quality Management cohort were included. Effectiveness of treatment at 1 year, as well as drug survival, was compared between subgroups having discontinued the first TNFi because of lack of response, adverse events (AEs), or other reasons. Lack of response was further divided into primary or secondary lack of response (PLR or SLR, respectively), depending on whether the first TNFi was stopped before or after 6 months of treatment.

**Results:**

Among 632 patients with axSpA, median survival of a second TNFi was 1.1 years after PLR and 3.8 years after SLR (*p* = 0.003). At least moderate disease activity as defined by an Ankylosing Spondylitis Disease Activity Score using the erythrocyte sedimentation rate (ASDAS-ESR) <2.1 was achieved after 12 months by 11 %, 39 %, 26 %, and 39 % of patients who discontinued their first TNFi because of PLR, SLR, AEs, and other reasons, respectively (*p* = 0.01). Only 4 % of patients achieved an ASDAS-ESR inactive disease state after PLR, in comparison to 22 % of those after SLR. Similar results were demonstrated in patients fulfilling the Assessment of SpondyloArthritis international Society classification criteria for axSpA (*n* = 488): ASDAS-ESR <2.1 was achieved after 12 months by 9 %, 41 %, 29 %, and 39 % of patients who discontinued their first TNFi because of PLR, SLR, AEs, and other reasons, respectively (*p* = 0.01).

**Conclusions:**

The effectiveness of a second TNFi is significantly impaired in patients with axSpA after PLR to a first TNFi compared with SLR.

**Electronic supplementary material:**

The online version of this article (doi:10.1186/s13075-016-0969-2) contains supplementary material, which is available to authorized users.

## Background

Although the use of tumor necrosis factor-α inhibitors (TNFi) has revolutionized the treatment of axial spondyloarthritis (axSpA), a significant proportion of patients do not adequately respond [1–5]. Young age, male sex, high baseline Bath Ankylosing Spondylitis Disease Activity Index (BASDAI), low baseline Bath Ankylosing Spondylitis Functional Index (BASFI), high baseline C-reactive protein (CRP), human leukocyte antigen B27 (HLA-B27) positivity, and the absence of enthesitis have been described as predictors of good response to TNFi [6, 7]. We additionally identified smoking to be associated with a worse outcome following TNFi treatment in patients with axSpA [8]. Switching to an alternative TNFi appears to be associated with lower response and/or drug survival rates in patients with ankylosing spondylitis (AS) [9–24]. In patients with rheumatoid arthritis (RA), response to a second TNFi seemed better if the first TNFi was discontinued because of loss of efficacy or adverse events (AEs) in comparison to a primary lack of efficacy [25, 26]. So far, no evidence for a differential response to a second TNFi in dependence on the reason for discontinuation of the first TNFi has been observed in axSpA. As new compounds with different modes of action are currently being tested in axSpA as potential alternatives to TNFi switching [27–29], we explored the effectiveness of switching TNFi in a large real-life observational axSpA cohort.

## Methods

### Study population

We conducted a longitudinal analysis of data collected annually from patients with a clinical diagnosis of axSpA, including AS, recruited in the ongoing Swiss Clinical Quality Management (SCQM) Cohort from January 2005 to September 2015 [30]. Clinical assessments included a physical examination (spinal and hip mobility according to the Bath Ankylosing Spondylitis Metrology Index, measurement of height and weight, presence of peripheral arthritis, dactylitis as well as enthesitis), laboratory tests (erythrocyte sedimentation rate [ESR] and CRP levels), data on treatment with nonsteroidal anti-inflammatory drugs (NSAIDs) as present or absent, and data on conventional and biologic disease-modifying drugs with dosage and start and stop dates [31]. The following reasons for drug discontinuation were specified in the database by the treating rheumatologist: insufficient effectiveness, AEs, remission, and other reasons. There was no further specification of the “other reasons” category of discontinuation in the SCQM questionnaire, but these reasons may be manifold, such as personal preference by the patient or physician, pregnancy, or elective surgery. As discontinuation due to remission was observed in only 1.9 % of the patients, we pooled this category together with the “other reasons” category for discontinuation. Patient questionnaires included the BASDAI, the BASFI, smoking status (never, previous, or current), and the number of weekly exercise sessions.

### Inclusion criteria for the present study

Patients with a clinical diagnosis of axSpA who had initiated a second TNFi after recruitment into the SCQM-axSpA cohort were included. Interruptions of treatment with the same TNFi were not counted as switches. Patients with overlapping TNFi courses or with an unclear start date were excluded. The study was approved by the ethics commission of the Canton of Zurich. Written informed consent was obtained from all patients.

### Outcomes

The primary outcome was drug survival of a second TNFi in relation to the reason for discontinuation of the first TNFi. In the case of several reasons for drug discontinuation, the following hierarchy was implemented: lack of effect > AE > other reasons. Only the discontinuation reason highest in hierarchy was used. Lack of efficacy was further divided into primary lack of response (PLR) if the first TNFi was stopped within 6 months after start and in secondary lack of response (SLR) if the first TNFi was discontinued after a 6-month period. The Assessment of SpondyloArthritis international Society (ASAS) recommends assessment of response to treatment after at least 12 weeks [32]. We chose a cutoff of 6 months, however, as clinically relevant improvement may take longer than 3 months [33, 34]. Moreover, the treating rheumatologist has to apply for reimbursement for an alternative TNFi in Switzerland, which may delay the switching process for a couple of weeks. The co–primary outcome of interest was effectiveness of treatment, assessed as the proportion of patients reaching at least an Ankylosing Spondylitis Disease Activity Score (ASDAS) moderate disease activity state (ASDAS <2.1), an ASDAS inactive disease state (ASDAS <1.3), or the ASAS criteria for partial remission (ASAS-PR) at 12 ± 3 months [35, 36]. Achievement of ASDAS cutoffs is primarily presented using the ESR, as CRP levels are registered in SCQM Cohort with the respective reference level and not the detection level, thus impeding the recently proposed ASDAS-CRP imputation [37] in some patients. Results derived using ASDAS-CRP are presented in (see Additional file 2: Table S2) after assuming a constant number of 2 for CRP levels <2. Response was assessed in patients with available outcome values at 12 months. Patients with available outcome measures at this time point who had discontinued the first TNFi but had not started an alternative TNFi were considered nonresponders (response/tolerance analysis). Additionally, response was assessed only among patients still on treatment at 12 ± 3 months (per-protocol response analysis).

### Statistical analysis

Baseline characteristics in terms of categorical variables were compared between patients starting a second TNFi after different reasons for discontinuation of the first TNFi using the χ^2^ test. For symmetrically distributed discrete or continuous variables, analysis of variance was used for testing whether the means in the different groups were equal. The Kruskal-Wallis test was used for data with skewed distribution. All tests were two-sided, with the significance level set at 0.05.

Drug maintenance was described with Kaplan-Meier plots. The log-rank test was used for testing differences between groups shown in the plots. Multiple adjusted Cox proportional hazards models were set up to estimate a covariate-adjusted effect of the reason for discontinuation of the first TNFi on the drug maintenance of the second TNFi. Ongoing treatments were censored at the last visit in the cohort. The following covariates were used: sex, age, calendar year of switch (to account for the number of various anti-TNF agents available for switch), the individual anti-TNF agents, and the type of TNFi switch (monoclonal antibody [mAb] to mAb versus mAb to soluble receptor anti-TNF agent and vice versa). To assess the significance of differences in response rates after 1 year of treatment with the second TNFi, Fisher’s exact test was used. R statistical software was used for all analyses.

## Results

### Patient disposition and baseline characteristics

A total of 686 patients with axSpA started treatment with a second TNFi after inclusion in the SCQM Cohort. A total of 54 patients lost to follow-up after the start of a second TNFi were excluded from the analyses. The baseline characteristics at the start of the second TNFi in these patients, stratified by the reason of discontinuation of the first TNFi (PLR in 23.1 %, SLR in 42.7 %, AEs in 19.8 %, other in 14.4 %), are shown in Table 1. There was an enrichment of patients with predictors of an impaired response to TNFi in the group having stopped the first TNFi because of PLR: higher proportion of HLA-B27 negativity and presence of enthesitis, higher BASFI, and higher proportions of smokers and of patients classified as having nonresponsive axSpA. Patients in the PLR group also displayed higher baseline BASDAI and ASDAS levels, and a higher percentage were treated with NSAIDs. A similar enrichment of patients with predictors of an unfavorable response, as well as of patients with a higher disease activity, was found in patients who met the ASAS classification criteria for axSpA (*n* = 488) (Table 2). The proportion of patients stopping their first TNFi because of PLR was similar in the groups with a clinical diagnosis of axSpA, those fulfilling the ASAS criteria, and those who met the modified New York criteria (23.1 %, 22.5 %, and 20.0 %, respectively). A similar proportion of TNFi-treated patients in the PLR and SLR groups was concurrently treated with conventional disease-modifying antirheumatic drugs (Tables 1 and 2).

**Table 1:** Characteristics of patients with a clinical diagnosis of axial spondyloarthritis starting a second tumor necrosis factor inhibitor

Parameter	Number of patients	PLR (*n* = 146)	SLR (*n* = 270)	AE (*n* = 125)	Other (*n* = 91)	*p* Value
Male sex, %	632	47.3	56.3	46.4	52.8	0.20
Age, years	632	43.8 ± 10.5	44.4 ± 11.1	44.1 ± 12.3	45.0 ± 13.1	0.87
Radiographic axSpA, %	454	54.4	69.2	65.8	82.1	0.003
HLA-B27–positive, %	510	43.2	67.1	64.3	71.0	<0.001
Elevated CRP, %	361	30.4	34.2	42.7	29.2	0.33
ASDAS-CRP	316	3.4 ± 0.8	3.1 ± 0.9	3.0 ± 1.1	2.7 ± 1.0	<0.001
ASDAS-ESR	289	3.1 ± 0.8	2.8 ± 0.8	2.8 ± 1.2	2.6 ± 1.2	0.03
BASDAI	348	6.0 ± 1.8	5.2 ± 2.0	4.9 ± 2.3	4.5 ± 2.3	<0.001
BASFI	345	4.6 ± 2.5	4.1 ± 2.5	3.7 ± 2.5	2.8 ± 2.3	0.001
BASMI	288	2.1 ± 1.9	2.3 ± 2.0	2.4 ± 2.1	2.7 ± 1.9	0.36
Peripheral arthritis, %	629	41.4	37.0	31.7	30.8	0.26
Enthesitis heel, %	632	42.5	31.9	26.4	24.2	0.01
Smokers, %	602	67.6	57.2	69.0	50.0	0.01
DMARDs, %	632	21.2	24.1	12.0	24.2	0.03
NSAIDs, %	632	62.3	54.4	48.0	39.6	0.003
BMI, kg/m^2^	608	26.3 ± 4.9	25.6 ± 4.9	25.9 ± 4.7	25.7 ± 4.7	0.58
Years of education	596	13.0 ± 3.0	13.5 ± 3.0	13.3 ± 2.9	14.1 ± 3.2	0.07

*axSpA* axial spondyloarthritis, *ASAS* Assessment of SpondyloArthritis international Society, *ASDAS-CRP* Ankylosing Spondylitis Disease Activity Score using C-reactive protein, *ASDAS-ESR* Ankylosing Spondylitis Disease Activity Score using erythrocyte sedimentation rate, *BASDAI* Bath Ankylosing Spondylitis Disease Activity Index, *BASFI* Bath Ankylosing Spondylitis Functional Index, *BASMI* Bath Ankylosing Spondylitis Metrology Index, *DMARDs* disease-modifying antirheumatic drugs, *NSAIDs* nonsteroidal anti-inflammatory drugs, *BMI* body mass index

Except where indicated otherwise, values for continuous variables are mean (±SD)

**Table 2:** Characteristics of patients fulfilling the ASAS axial spondyloarthritis classification criteria starting a second tumor necrosis factor inhibitor

Parameter	Number of patients	PLR (*n* = 110)	SLR (*n* = 220)	AE (*n* = 88)	Other (*n* = 70)	*p* Value
Male sex, %	488	47.3	60.9	53.4	55.7	0.12
Age, years	488	42.8 ± 10.2	43.2 ± 10.9	42.3 ± 11.8	44.2 ± 13.4	0.77
Radiographic axSpA, %	388	66.7	77.4	82.5	87.9	0.02
HLA-B27–positive, %	442	51.6	73.7	75.0	78.3	<0.001
Elevated CRP, %	285	34.8	37.0	52.0	30.8	0.15
ASDAS-CRP	256	3.4 ± 0.9	3.1 ± 0.9	3.0 ± 1.2	2.7 ± 1.1	0.003
ASDAS-ESR	233	3.2 ± 0.8	2.8 ± 0.8	2.8 ± 1.2	2.7 ± 1.2	0.08
BASDAI	275	6.1 ± 1.9	5.1 ± 2.0	4.7 ± 2.4	4.6 ± 2.3	<0.001
BASFI	274	4.7 ± 2.5	4.1 ± 2.5	3.5 ± 2.4	3.0 ± 2.2	0.002
BASMI	228	2.1 ± 1.7	2.3 ± 2.0	2.7 ± 2.3	2.9 ± 2.0	0.27
Peripheral arthritis, %	487	36.7	35.5	26.1	21.4	0.06
Enthesitis heel, %	488	40.0	33.2	26.1	22.9	0.06
Smokers, %	471	73.4	58.4	72.3	55.7	0.01
DMARDs, %	488	20.0	22.7	9.1	17.1	0.03
NSAIDs, %	488	62.7	54.5	50.0	35.7	0.01
BMI, kg/m^2^	478	26.1 ± 4.6	25.7 ± 4.8	26.0 ± 4.7	26.2 ± 4.8	0.88
Years of education	463	13.0 ± 3.2	13.5 ± 3.0	13.0 ± 2.9	13.9 ± 3.2	0.23

*axSpA* axial spondyloarthritis, *ASAS* Assessment of SpondyloArthritis international Society, *ASDAS-CRP* Ankylosing Spondylitis Disease Activity Score using C-reactive protein, *ASDAS-ESR* Ankylosing Spondylitis Disease Activity Score using erythrocyte sedimentation rate, *BASDAI* Bath Ankylosing Spondylitis Disease Activity Index, *BASFI* Bath Ankylosing Spondylitis Functional Index, *BASMI* Bath Ankylosing Spondylitis Metrology Index, *DMARDs* disease-modifying antirheumatic drugs, *NSAIDs* nonsteroidal anti-inflammatory drugs, *BMI* body mass index

Except where indicated otherwise, values for continuous variables are mean (±SD)

### Drug retention

**Fig. 1:**
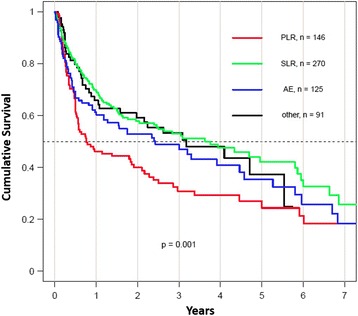
Drug survival of the second tumor necrosis factor inhibitor (TNFi), stratified by the reason for discontinuation of the first TNFi, in patients with a clinical diagnosis of axial spondyloarthritis. *Other* refers to reason for discontinuation other than lack of effect or intolerance. *AE* adverse events, *PLR* primary lack of response, *SLR* secondary lack of response

**Fig. 2:**
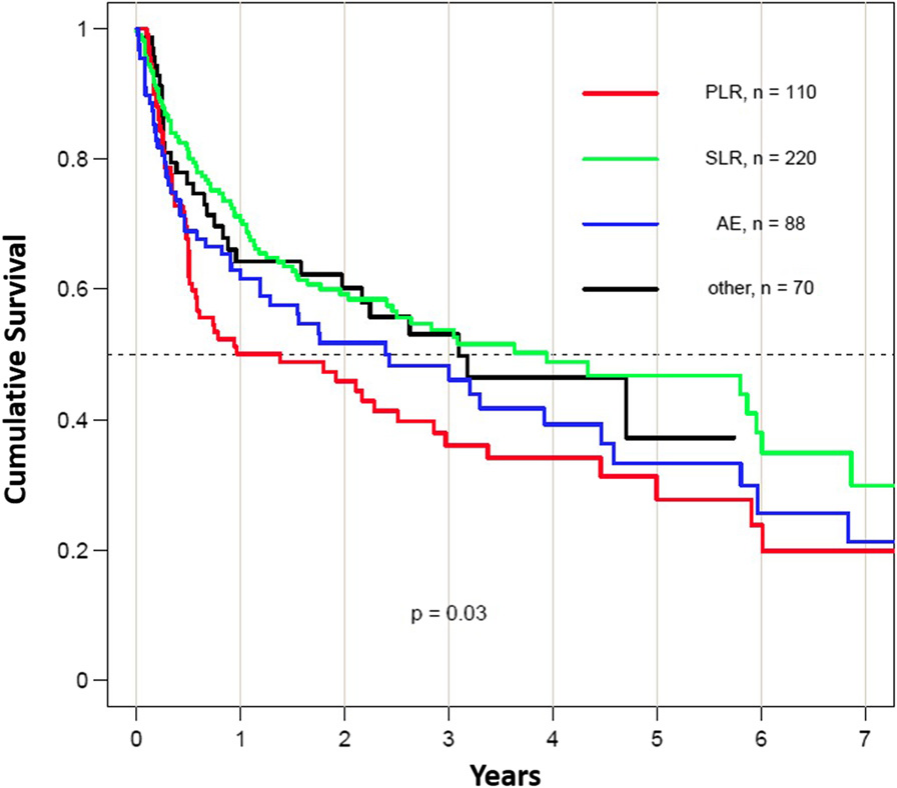
Drug survival of the second tumor necrosis factor inhibitor (TNFi), stratified by the reason for discontinuation of the first TNFi, in patients fulfilling the Assessment of SpondyloArthritis international Society axial spondyloarthritis classification criteria. *Other* refers to reason for discontinuation other than lack of effect or intolerance. *AE* adverse events, *PLR* primary lack of response, *SLR* secondary lack of response

The median drug retention of the second TNFi was 2.29 years (95 % confidence interval [CI] 1.79–2.97) for all patients with axSpA and 2.61 years (95 % CI 2.05–3.28) in the subgroup fulfilling the ASAS axSpA classification criteria. Drug maintenance depending on the reason for discontinuation of the first TNFi is shown in Fig. 1 for all patients with a clinical diagnosis of axSpA and in Fig. 2 for patients fulfilling the ASAS axSpA classification criteria. Significant differences in retention rates were found between the four groups (*p* = 0.001), with the shortest drug survival observed after previous PLR. The median drug survival of a second TNFi was 1.06 years (95 % CI 0.75–1.96) after PLR and 3.76 years (95 % CI 3.12–4.28) after SLR (*p* = 0.003). This difference remained significant after adjustment for sex, age, the calendar year of switching (reflecting the number of available TNFi at each time point), and the type of TNFi switching (mAb to mAb versus mAb to fusion protein anti-TNF agent and vice versa) (Table 3). The hazard ratio for discontinuing the second TNFi after previous SLR in comparison to PLR was 0.56 (95 % CI 0.42–0.75, *p* < 0.001) in all patients diagnosed as having axSpA and 0.58 (95 % CI 0.42–0.81, *p* = 0.002) in those patients fulfilling the ASAS axSpA classification criteria. Similar results were found after replacing the type of TNFi switching by the various anti-TNF agents in the model (adalimumab, certolizumab, etanercept, golimumab, infliximab).

**Table 3:** Cox models for drug retention of a second tumor necrosis factor inhibitor in patients having discontinued the first tumor necrosis factor inhibitor due to primary or secondary lack of response

	Patients with a clinical diagnosis of axSpA^a^			Patients fulfilling the ASAS axSpA classification criteria^b^		
	HR	95 % CI	*p* Value	HR	95 % CI	*p* Value
SLR vs. PLR	0.56	0.42–0.75	<0.001	0.58	0.42–0.81	0.002
Female vs. male	0.94	0.71–1.24	0.65	0.90	0.64–1.25	0.53
Age	1.00	0.99–1.02	0.73	1.00	0.99–1.02	0.93
Cept → mAb^c^	1.11	0.79–1.55	0.56	1.17	0.78–1.73	0.45
mAb → Cept^c^	1.11	0.80–1.56	0.54	1.24	0.84–1.82	0.28
2007–2010 vs. before 2006	1.26	0.50–3.12	0.63	1.18	0.47–2.96	0.73
2010–2015 vs. before 2006	1.58	0.63–3.99	0.33	1.44	0.57–3.67	0.44

*ASAS* Assessment of SpondyloArthritis international Society, *axSpA* axial spondyloarthritis, *Cept* soluble receptor anti–tumor necrosis factor agent, *mAb* monoclonal antibody anti–tumor necrosis factor agent, *HR* hazard ratio, *CI* confidence Interval, *PLR* primary lack of response, *SLR* secondary lack of response

^a^Total of 416 patients and 207 discontinuation events

^b^Total of 330 patients and 157 discontinuation events

^c^Indicated switch type versus reference category mAb → mAb

### Clinical response

Response to treatment with a second TNFi was assessed in patients with available outcome values at 12 ± 3 months (ASAS-PR [*n* = 227, 36 %] and ASDAS-ESR [*n* = 184, 29 %]). Patients with versus without follow-up at this time point did not differ with regard to important baseline disease characteristics (BASDAI, ASDAS, elevated CRP, HLA-B27, classification as AS, age, peripheral arthritis, enthesitis, smoking, education, reason for discontinuation of first TNFi, physical exercise, body mass index) (see Additional file 1: Table S1).

Response rates to a second TNFi differed significantly between the subgroups, based on the reasons for discontinuation of the first TNFi (Table 4). These were most impaired in the subgroup of patients having discontinued the first TNFi as a consequence of PLR, followed by AEs. At least moderate disease activity (defined by ASDAS-ESR <2.1) was reached by 11 %, 26 %, and 39 % of patients in the PLR, AE, and SLR groups, respectively. Only a negligible proportion of patients achieved ASAS-PR or ASDAS-ESR inactive disease state after PLR (2 % and 4 % of patients, respectively), as opposed to 13 % and 22 %, respectively, after SLR. Similar results were found in patients fulfilling the ASAS axSpA classification criteria (Table 4) and when using the CRP for ASDAS calculation (see Additional file 2: Table S2).

**Table 4:** Response rates after 1 year of treatment with a second tumor necrosis factor inhibitor, stratified by the reason of discontinuation of the first tumor necrosis factor inhibitor

Response criterion	Analysis	Number of patients	All	PLR	SLR	AE	Other	*p* Value^a^	*p* Value^b^
Patients with a clinical diagnosis of axSpA									
ASDAS-ESR <2.1	Response/tolerance	184	28.8	11.1	38.9	26.2	38.9	0.01	0.002
ASDAS-ESR <2.1	Per protocol	124	42.7	21.7	50.8	35.5	63.6	0.04	0.02
ASDAS-ESR <1.3	Response/tolerance	184	14.7	4.4	21.5	9.5	22.2	0.03	0.01
ASDAS-ESR <1.3	Per protocol	124	21.8	8.7	28.8	12.9	36.4	0.07	0.08
ASAS-PR	Response/tolerance	227	11.0	2.0	12.5	13.7	16.7	0.08	0.04
ASAS-PR	Per protocol	146	17.1	4.2	17.4	18.4	33.3	0.12	0.17
Patients fulfilling the ASAS axSpA classification									
ASDAS-ESR <2.1	Response/tolerance	148	31.1	9.1	40.9	29.0	38.9	0.01	0.001
ASDAS-ESR <2.1	Per protocol	100	46.0	18.8	55.1	37.5	63.6	0.04	0.02
ASDAS-ESR <1.3	Response/tolerance	148	16.2	3.0	22.7	12.9	22.2	0.045	0.02
ASDAS-ESR <1.3	Per protocol	100	24.4	6.2	30.6	16.7	36.4	0.13	0.09
ASAS-PR	Response/tolerance	179	11.7	0.0	12.5	16.7	18.5	0.03	0.03
ASAS-PR	Per protocol	106	18.1	0.0	17.5	21.4	35.7	0.05	0.10

*axSpA* axial spondyloarthritis, *ASDAS-CRP* Ankylosing Spondylitis Disease Activity Score using C-reactive protein, *ASDAS-ESR* Ankylosing Spondylitis Disease Activity Score using erythrocyte sedimentation rate, *AE* adverse events, *PLR* primary lack of response, *SLR* secondary lack of response

*Other* refers to reason of discontinuation other than lack of effect or intolerance. *Response/tolerance* refers to proportion of patients with a valid follow-up achieving the respective response criterion (with patients having discontinued treatment being defined as nonresponders). *Per protocol* refers to proportion of patients achieving the respective response criterion among those patients still receiving treatment

Except where indicated otherwise, values are percentages

^a^ *p* Value overall

^b^ *p* Value PLR vs. SLR

## Discussion

Our TNFi switching study in axSpA, which to our knowledge is the largest so far, suggests that the reason for discontinuation of a first TNFi may affect the effectiveness of a second TNFi, as previously reported in RA [25, 26]. Drug retention and treatment responses after switching to a second TNFi in axSpA were impaired in patients having discontinued the first TNFi due to primary lack of effectiveness in comparison to SLR. Earlier investigations had been hampered by the fact that it was often not possible to distinguish between these two reasons for drug discontinuation [18, 22]. As ASAS recommends assessment of treatment response after at least 12 weeks [32] but time to improvement may be longer than 3 months [33, 34], we have defined a discontinuation due to an insufficient effect after 6 months of treatment as being the consequence of a loss of efficacy. This cutoff allowed us to evaluate drug retention and response rates of the second TNFi. We found a difference of 2.7 years in median retention of the second TNFi between patients in the PLR and SLR groups. Moreover, an ASDAS-ESR inactive disease state was reached by only 4 % of patients after previous PLR in comparison to 22 % after SLR. Thus, PLR may identify a subgroup of patients in whom TNF probably does not play a major role in disease pathogenesis and amplification of inflammation. Whether these patients would experience a superior response to biologics with a different mode of action, as demonstrated for RA [38], remains to be established. An alternative, though mutually not exclusive, reason for impaired effectiveness of the second TNFi in the PLR group is a higher proportion of patients with predictors of an impaired response to anti-TNF agents [7] in our study (normal CRP, HLA-B27 negativity, high BASFI levels, and frequent enthesitis). Furthermore, misdiagnosis in some patients in the PLR group cannot be ruled out in the PLR group, given the low percentage of HLA-B27–positive patients (43 %). We expected a lower proportion of PLR patients in the groups fulfilling the ASAS classification criteria or the modified New York classification criteria, which was not the case, however. Finally, even with a correct diagnosis of axSpA, patients may have additional reasons for back pain (e.g., degenerative spinal disease or fibromyalgia [39–41]), which may be misinterpreted as axSpA activity, prompting the initiation of anti-TNF treatment. Reassessment of a diagnosis of axSpA and of musculoskeletal comorbidities in patients having experienced PLR to a first TNFi seems advisable before initiating a next biologic.

The results were very similar in patients with a clinical diagnosis of axSpA made by their treating rheumatologist and those fulfilling the ASAS classification criteria for axSpA. The presentation of patients with axSpA diagnosed on clinical grounds also allows comparison with TNFi switching in other observational cohorts [18, 22], where the proportion of patients fulfilling the modified New York criteria or the ASAS axSpA classification criteria was not reported. The median drug survival of a second TNFi was 2.3 years in SCQM and 1.6 years in the Danish DANBIO registry [22]. In DANBIO, the proportion of patients with a clinical diagnosis of AS treated for at least 2 years with a second TNFi who reached an ASDAS-CRP <2.1 was 37 %. We found a similar proportion of patients with axSpA (43 %) with an ASDAS-ESR <2.1 at 1 year of treatment with a second TNFi. Our result differed significantly in dependence on the reason for discontinuation of the first TNFi: only 22 % after previous PLR, 36 % after AEs and 51 % after SLR. Over 75 % of the patients in the SCQM Cohort met the ASAS classification criteria for axSpA. Patients fulfilling the ASAS criteria showed comparable response rates (with 19 %, 38 %, and 55 % reaching an ASDAS-ESR <2.1 in the PLR, AE, and SLR groups, respectively) to those of the patients in the entire cohort defined by the treating rheumatologists.

A limitation of our response analyses is that follow-up data of ASDAS at 12 months was available for only approximately one-third of patients. However, patients with versus without follow-up at this time point did not differ with regard to clinically relevant baseline disease characteristics. The limitation of incomplete follow-up is inherent to observational registries. In the NOR-DMARD registry [18], ASDAS responses were available in 25 % of patients at 3 months and 29 % at the last observation and in DANBIO in 45 % of patients at 2 years [22]. There was no difference in response rates among the various anti-TNF agents used as second-line treatment in DANBIO, while drug survival of second treatment courses was longer in adalimumab-treated patients previously treated with infliximab [22]. In RA, drug survival of infliximab was shown to be inferior to adalimumab or etanercept [26], although other comparisons yielded a similar maintenance of various TNFi [42]. Moreover, the type of TNFi switch was shown to affect the effectiveness of a second TNFi in RA: Switching from an anti-TNF mAb to a soluble TNF receptor yielded better results than vice versa [26]. We were unable to confirm differences between individual anti-TNF agents or between various types of switching with regard to drug maintenance.

## Conclusions

Our findings suggest that the effectiveness of a second TNFi is impaired in patients with axSpA who have experienced a PLR to a first TNFi during the first 6 months of treatment.

## Additional files

Additional file 1: Table S1. Comparison of baseline characteristics of patients with and without a follow-up at 1 year of treatment. (DOC 38 kb) Additional file 2: Table S2. ASDAS-CRP response rates after 1 year of treatment with a second TNFi, stratified by the reason for discontinuation of the first TNF inhibitor. (DOC 38 kb)

## Abbreviations

AE: adverse event
AS: ankylosing spondylitis
ASAS: Assessment of SpondyloArthritis international Society
ASDAS: Ankylosing Spondylitis Disease Activity Score
axSpA: axial spondyloarthritis
BASDAI: Bath Ankylosing Spondylitis Disease Activity Index
BASFI: Bath Ankylosing Spondylitis Functional Index
BASMI: Bath Ankylosing Spondylitis Mobility Index
BMI: body mass index
CI: confidence interval
CRP: C-reactive protein
DMARD: disease-modifying antirheumatic drug
ESR: erythrocyte sedimentation rate
HLA-B27: human leukocyte antigen B27
HR: hazard ratio
mAb: monoclonal antibody
NSAID: nonsteroidal anti-inflammatory drug
PLR: primary lack of response
PR: partial remission
RA: rheumatoid arthritis
SCQM: Swiss Clinical Quality Management
SLR: secondary lack of response
TNFi: tumor necrosis factor inhibitor
