# Supplementary material for: Does the reason for discontinuation of a first TNF inhibitor influence the effectiveness of a second TNF inhibitor in axial spondyloarthritis? Results from the Swiss Clinical Quality Management Cohort
Source: Arthritis Res Ther. 2016 Mar 22;18:71. doi: 10.1186/s13075-016-0969-2 (PMC4802885; doi:10.1186/s13075-016-0969-2)
Supplement: Additional file 1: Table S1. — Comparison of baseline characteristics of patients with and without a follow-up at 1 year of treatment. (DOC 38 kb) [file 13075_2016_969_MOESM1_ESM.doc]

**Table S1. Comparison of baseline characteristics of patients with and**

without a follow-up at 1 year of treatment

| **Parameter** | **N** | **With**  **Follow-up** | **Without**  **Follow-up** | **p** |
| --- | --- | --- | --- | --- |
| Age, years | 632 | 45.2±12.0 | 44.0±11.2 | 0.24 |
| Radiographic axSpA, % | 464 | 68.2 | 66.1 | 0.63 |
| HLA-B27 positive, % | 552 | 63.0 | 61.5 | 0.77 |
| Elevated CRP, % | 361 | 36.8 | 33.0 | 0.49 |
| ASDAS | 289 | 2.9±0.9 | 2.9±1.0 | 0.53 |
| BASDAI | 348 | 5.5±2.1 | 5.1±2.2 | 0.15 |
| Peripheral arthritis, % | 629 | 35.5 | 36.3 | 0.93 |
| Enthesitis heel, % | 632 | 35.9 | 30.6 | 0.22 |
| Smokers, % | 602 | 62.6 | 60.2 | 0.59 |
| DMARDs, % | 632 | 21.7 | 20.8 | 0.83 |
| NSAIDs, % | 632 | 57.1 | 51.1 | 0.19 |
| BMI | 608 | 26.0±4.7 | 25.8±4.9 | 0.63 |
| Years of Education | 596 | 13.6±2.8 | 13.3±3.2 | 0.32 |
| Reason of discontinuation of first TNFi: adverse events | 632 | 11.4 | 12.1 | 0.89 |
| Reason of discontinuation of first TNFi: lack of response | 632 | 34.8 | 31.7 | 0.46 |

Except where indicated otherwise, values for continuous variables are the mean (±SD). ASDAS = Ankylosing Spondylitis Disease Activity Score; BASDAI = Bath Ankylosing Spondylitis Disease Activity Index; DMARDs = Disease Modifying Anti-Rheumatic Drugs; NSAIDs = Non-Steroidal Anti-Inflammatory Drugs; BMI = Body Mass Index; TNFi = Tumour Necrosis Factor Inhibitor
