# Supplementary material for: Does the reason for discontinuation of a first TNF inhibitor influence the effectiveness of a second TNF inhibitor in axial spondyloarthritis? Results from the Swiss Clinical Quality Management Cohort
Source: Arthritis Res Ther. 2016 Mar 22;18:71. doi: 10.1186/s13075-016-0969-2 (PMC4802885; doi:10.1186/s13075-016-0969-2)
Supplement: Additional file 2: Table S2. — ASDAS-CRP response rates after 1 year of treatment with a second TNFi, stratified by the reason for discontinuation of the first TNF inhibitor. (DOC 38 kb) [file 13075_2016_969_MOESM2_ESM.doc]

**Table S2**. ASDAS-CRP response rates after 1 year of treatment with a second TNFi, stratified by the reason of discontinuation of the first TNFi

| **A. Patients with a clinical diagnosis of axSpA** | | | | | | | | | |
| --- | --- | --- | --- | --- | --- | --- | --- | --- | --- |
| **Response criterion** | **Analysis** | **N** | **All** | **PLR** | **SLR** | **AE** | **Other** | **P*** | **P**** |
| **ASDAS-CRP <2.1** | Response/tolerance | 190 | 23.7 | 8.7 | 29.8 | 19.5 | 42.1 | 0.01 | 0.01 |
| **ASDAS-CRP <2.1** | Per protocol | 128 | 35.2 | 17.4 | 39.7 | 35.8 | 72.7 | 0.01 | 0.07 |
| **ASDAS-CRP <1.3** | Response/tolerance | 190 | 7.9 | 2.2 | 11.9 | 2.4 | 15.8 | 0.06 | 0.10 |
| **ASDAS-CRP <1.3** | Per protocol | 128 | 11.7 | 4.3 | 15.9 | 3.2 | 27.3 | 0.07 | 0.27 |
| **B. Patients fulfilling the ASAS axSpA classification** | | | | | | | | | |
| **Response criterion** | **Analysis** | **N** | **All** | **PLR** | **SLR** | **AE** | **Other** | **P*** | **P**** |
| **ASDAS-CRP < 2.1** | Response/tolerance | 150 | 29.9 | 9.1 | 33.8 | 23.3 | 42.1 | 0.02 | 0.01 |
| **ASDAS-CRP <2.1** | Per protocol | 102 | 44.4 | 18.8 | 45.1 | 29.2 | 72.7 | 0.02 | 0.01 |
| **ASDAS-CRP <1.3** | Response/tolerance | 150 | 15.6 | 3.0 | 13.2 | 0.0 | 15.8 | 0.05 | 0.16 |
| **ASDAS- CRP < 1.3** | Per protocol | 102 | 32.2 | 6.2 | 17.6 | 0.0 | 27.3 | 0.03 | 0.43 |

Except where indicated otherwise, values are the percent. TNFi = tumor necrosis factor inhibitor; axSpA = axial spondyloarthritis; ASDAS-ESR = Ankylosing Spondylitis Disease Activity Score using the erythrocyte sedimentation rate; AE = adverse events; PLR = primary lack of response; SLR = secondary lack of response; other = reason of discontinuation other than lack of effect or intolerance. Response/tolerance-Analysis = Proportion of patients with a valid follow-up achieving the respective response criterion (patients having discontinued treatment being defined as non-responders); Per-protocol-analysis = Proportion of patients achieving the respective response criterion out of those patients still on treatment. * p value overall; ** p value PLR vs. SLR.
